# Predicting the risk of clinically significant intraocular lens tilt and decentration in vitrectomized eyes

**DOI:** 10.1097/j.jcrs.0000000000000997

**Published:** 2022-06-27

**Authors:** Jiaqing Zhang, Xiaotong Han, Miao Zhang, Zhenzhen Liu, Xiaoyun Chen, Xiaozhang Qiu, Haowen Lin, Jiaqing Li, Bingqian Liu, Chun Zhang, Yantao Wei, Guangming Jin, Xuhua Tan, Lixia Luo

**Affiliations:** From the State Key Laboratory of Ophthalmology, Zhongshan Ophthalmic Center, Sun Yat-sen University, Guangdong Provincial Key Laboratory of Ophthalmology and Visual Science, Guangzhou, China (J. Zhang, Han, M. Zhang, Z. Liu, Chen, Qiu, Lin, Li, B. Liu, C. Zhang, Wei, Jin, Tan, Luo); Guangdong Provincial Clinical Research Center for Ocular Diseases, Guangzhou, China. (J. Zhang, Han, M. Zhang, Z. Liu, Chen, Qiu, Lin, Li, B. Liu, C. Zhang, Wei, Jin, Tan, Luo).

## Abstract

To identify predictors and develop a prognostic nomogram for clinically significant intraocular lens (IOL) tilt and decentration in vitrectomized eyes.

**Setting:**

Zhongshan ophthalmic center, Guangzhou, China.

**Design:**

Prospective observational study.

**Methods:**

Patients with previous pars plana vitrectomy who underwent phacoemulsification with IOL implantation were enrolled in this study. The tilt and decentration of the lens and IOL were assessed by a swept-source anterior segment optical coherence tomography (CASIA2). Multiple logistic regression analysis and prognostic nomogram models were used to explore factors associated with clinically significant IOL tilt and decentration (defined as tilt ≥7 degrees and decentration ≥0.4 mm).

**Results:**

375 patients (375 eyes) with a mean age of 56.1 ± 9.81 years were included. Lens tilt (odds ratio [OR] = 1.44), lens decentration (OR = 1.74), lens diameter (OR = 0.49), and hydrophilic IOL (OR = 2.36) were associated with IOL tilt over 7 degrees (all *P* < .05). Lens tilt (OR = 1.24), lens decentration (OR = 2.30), and incomplete capsulorhexis–IOL overlap (OR = 2.44) increased the risk of IOL decentration over 0.4 mm (all *P* < .05). Preoperative lens tilt together with lens decentration was identified as the strongest predictor of incident clinically significant IOL tilt (area under the curve [AUC] = 0.82, 95% CI, 0.76-0.88) and decentration (AUC: 0.84, 95% CI, 0.78-0.89), and the nomogram was constructed accordingly.

**Conclusions:**

The tilt and decentration of the crystalline lens, hydrophilic IOL, and incomplete capsulorhexis–IOL overlap were risk factors for clinically significant IOL misalignment. Clinicians could use a prognostic nomogram model based on the preoperative lens position to make a strategy for higher-risk patients.

## Abstract 2

Preoperative crystalline lens tilt and decentration showed strong predictive ability for postoperative clinically significant tilt and decentration of the intraocular lens in vitrectomized eyes.

Cataract formation or progression is inevitable after pars plana vitrectomy (PPV), and most patients require subsequent cataract surgery within 2 years after PPV.^1–3^ With the continuous advancement of vitreoretinal surgical techniques, the patients' expectations are also increasing.^4^ Precise alignment of the intraocular lens (IOL) with the visual axis is a prerequisite for good visual quality after cataract surgery. It has been proved in many studies that IOL tilt and decentration, especially with tilt over 7 degrees or decentration over 0.4 mm, could significantly impair postoperative visual function.^5–7^

We have previously reported that the risk of IOL misalignment was higher in vitrectomized eyes, approximately 20% of patients with cataract with prior PPV showed clinically significant IOL tilt (over 7 degrees) or decentration (over 0.4 mm), which was positively associated with larger internal higher-order aberrations.^8,9^ Given that the risk of IOL tilt or decentration could be minimized by the appropriate selection of the IOL type and use of capsular tension ring, the ability to identify patients with a high risk of postoperative IOL tilt or decentration in advance could better guide clinical decision and improve patient prognosis.^10,11^ However, to our knowledge, there is a lack of evidence regarding potential risk factors and their predictive ability for IOL misalignment for these patients.

The second-generation swept-source anterior segment optical coherence tomography (SS-ASOCT) (CASIA2, Tomey Corp.) can automatically quantify the IOL tilt and decentration relative to the corneal topographic axis in a 3D manner with high accuracy and repeatability.^8,12^ In this study, we aimed to identify clinical risk factors and further construct a prognostic nomogram for clinically significant IOL tilt and decentration in patients with cataract with prior PPV, based on measurements using CASIA2.

## METHODS

This prospective observational study was performed in accordance with the tenets of the Declaration of Helsinki and approved by the Institutional Review Board of the Zhongshan Ophthalmic Center in Sun Yat-sen University (No. 2019KYPJ033). Informed consent was obtained from all patients.

### Participants

Patients who planned to undergo phacoemulsification with IOL implantation after PPV surgery were consecutively recruited from March 1, 2021, to December 30, 2021, at Zhongshan Ophthalmic Center, Sun Yat-sen University, Guangzhou, China. Patients with any evidence of the following conditions were excluded: (1) previous history of scleral buckling; (2) iatrogenic crystalline lens injury during PPV; (3) comorbidities such as keratopathy, glaucoma, uveitis, ocular trauma, pseudoexfoliation syndrome, or lens dislocation; (4) undergoing secondly silicone oil (SO) tamponade due to recurrence of original disease; (5) intraoperative and postoperative complications, such as posterior capsule rupture, zonular dialysis, capsule contraction syndrome, or capsular block syndrome, or a combination of additional surgical procedure, such as posterior capsulotomy; and (6) incomplete follow-up information. If both eyes of the same patient meet the inclusion criteria, only the right eye is included for analysis, considering the significant intereye correlation.^13^

Post-PPV patients were divided into 3 subgroups. Patients in Group 1 had sequential cataract surgery without a history of SO tamponade; patients in Group 2 underwent sequential SO removal and cataract surgery; patients in group 3 had simultaneous SO removal and cataract surgery. All patients in our study used 5000-centistoke SO.

### Data Collection

Patient data, including age, sex, disease and surgical history, corrected distance visual acuity (CDVA) before cataract surgery, and surgical information (including the type and power of the implanted IOL), were collected from the electronic medical record. Preoperative ocular biometric parameters were measured by IOLMaster 700 (1.80, Carl Zeiss Meditec AG), including axial length (AL), keratometry, anterior chamber depth (ACD; measured from the epithelium to the lens), lens thickness (LT), and corneal diameter.

At 3 months or later after cataract surgery, all patients underwent comprehensive ocular examinations. The naked visual acuity and CDVA were measured at a distance of 4 meters with an Early Treatment Diabetic Retinopathy Study visual chart (Precision Vision). The slitlamp biomicroscope (BQ-900, Haag-Streit AG) and the 90 D lens were used to evaluate the anterior and posterior segments. The noncontact tonometer (CT-1 Computerized Tonometer, Topcon Corp.) was used for intraocular pressure measurements, and the mean value for 3 consecutive measurements was recorded. The standard digital retroillumination image under mydriatic conditions was taken to assess the capsulorhexis–IOL overlap.

### SS-OCT Imaging

All patients underwent anterior segment imaging by CASIA2 before and at 3 months or later after the cataract surgery. All measurements were taken by the same technician in a darkroom under mydriatic conditions. The lens biometry and IOL scan modes were used to measure the positions of crystalline lenses and IOLs, respectively. For crystalline lenses, CASIA2 produced 16 SS-OCT images of 16 scanning axes and generated a 3D image; then, the built-in software (v. SS2000) provided parameters, including the radius of curvature for the anterior and posterior surface of the lens, LT, tilt and decentration relative to the corneal topographic axis, and lens equatorial diameter. Similarly, IOL position, including ACD and IOL tilt and decentration, was measured based on 8 AS-OCT images from 8 different angles. The automatically marked outline of crystalline lenses and IOLs by CASIA2 was manually rechecked for accuracy. Images with severe artifacts were excluded including motion artifacts and data loss due to blinking.

### Statistical Analysis

The quantitative data were represented by mean ± SD or median with interquartile range (IQR) according to the normality of data examined by the Shapiro-Wilk test, and the qualitative data were displayed by frequency percentage. Accordingly, the one-way analysis of variance or the Kruskal-Wallis H test was performed to compare continuous variables among subgroups, and the chi-square test was used for categorical variables. Multiple comparisons were corrected by the Bonferroni method. The CDVA was recorded in decimal units and converted to the logMAR units.

Multiple logistic regression models were used to determine the associated factors of IOL tilt over 7 degrees and decentration over 0.4 mm. Factors with *P* < .05 in the univariate models were included in the multiple logistic regression model. To investigate the predictive ability of risk factors for incident IOL misalignment, receiver operating characteristic curves were plotted to calculate the area under the curve (AUC). Based on these analyses, prognostic nomogram models were constructed for IOL tilt over 7 degrees and decentration over 0.4 mm. The AUC and the Hosmer-Lemeshow goodness-of-fit test were used to depict the discrimination and calibration of the model, respectively. The model was internally validated using the 10-fold cross-validation method. All statistical analyses were performed with the commercially available software (Stata v. 16.0; Statacorp LLC; R v. 4.1.2, R Foundation for Statistical Computing). A *P* value less than 0.05 was considered statistically significant.

## RESULTS

**Figure 1.**
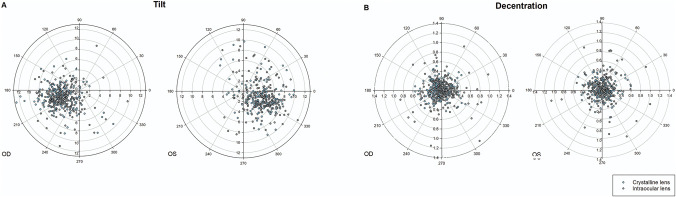
Polar coordinate graphs show the orientations and the values of the tilt and decentration. *A*: Lens and IOL tilt. *B*: Lens and IOL decentration.

In total, 375 eyes of 375 eligible patients (213 males [56.8%]) with a mean age of 56.1 ± 9.81 years were included. The demographic factors and preoperative ocular biometry of the study participants are listed in Table 1. The median AL was 24.5 mm (IQR: 23.4 to 26.3 mm), and 105 eyes (28%) had AL longer than 26 mm. The mean preoperative ACD was 3.11 ± 0.37 mm, and the mean LT was 4.55 ± 0.43 mm. The median lens tilt was 4.50 degrees (IQR: 3.40 to 6.20 degrees), and the median lens decentration was 0.18 mm (IQR: 0.11 to 0.28 mm). Most of the crystalline lens (72.3%) tilted toward the inferotemporal direction relative to the corneal topographic axis, whereas the directions of decentration showed no obvious tendency (Figure 1).

**Table 1.** Demographic and preoperative biometry of participants

Parameter	Total	Group 1	Group 2	Group 3
No. of eyes	375	121	79	175
Age (y), mean ± SD	56.1 ± 9.81	58.7 ± 10.6	52.1 ± 10.4	56.2 ± 8.29
Male, n (%)	213 (56.8)	59 (48.8)	46 (58.2)	108 (61.7)
Preop CDVA (logMAR), mean ± SD	1.22 ± 0.52	1.04 ± 0.50	1.37 ± 0.54	1.27 ± 0.50
Preop biometry				
AL (mm), median (IQR)	24.5 (23.4, 26.3)	24.2 (23.3, 26.3)	24.9 (23.8, 27.1)	24.2 (23.4, 26.0)
Mean K (D), mean ± SD	43.6 ± 1.58	43.6 ± 1.45	43.2 ± 1.59	43.8 ± 1.65
Corneal diameter (mm), mean ± SD	11.9 ± 0.46	11.9 ± 0.42	11.9 ± 0.42	11.9 ± 0.51
Preop ACD (mm), mean ± SD	3.11 ± 0.37	3.14 ± 0.40	3.10 ± 0.35	3.10 ± 0.35
RAL (mm), mean ± SD	9.41 ± 1.32	9.45 ± 1.44	9.11 ± 1.24	9.52 ± 1.25
RPL (mm), mean ± SD	5.81 ± 0.66	5.70 ± 0.54	5.99 ± 0.82	5.81 ± 0.65
LT (mm), mean ± SD	4.55 ± 0.43	4.59 ± 0.45	4.55 ± 0.46	4.53 ± 0.41
Lens vault (mm), mean ± SD	0.27 ± 0.31	0.27 ± 0.34	0.26 ± 0.32	0.28 ± 0.29
Lens tilt (°), median (IQR)	4.50 (3.40, 6.20)	4.30 (3.25, 5.40)	4.60 (3.20, 6.40)	4.90 (3.50, 6.60)
Lens decentration (mm), median (IQR)	0.18 (0.11, 0.28)	0.16 (0.10, 0.24)	0.21 (0.12, 0.33)	0.20 (0.12, 0.29)
Lens diameter (mm), mean ± SD	10.1 ± 0.59	10.0 ± 0.54	10.2 ± 0.72	10.0 ± 0.57

ACD = anterior chamber depth, as measured from the corneal epithelium to lens; AL = axial length; IQR = interquartile range; K = keratometry; LT = lens thickness; RAL = radius of anterior lens surface curvature; RPL = radius of posterior lens surface curvature

There were 121 eyes (32.2%) in group 1, 79 eyes (21.1%) in group 2, and 175 eyes (46.7%) in group 3. Patients in group 2 had younger age, and patients in group 1 showed better preoperative CDVA compared with the other 2 groups (both *P* < .001). The radius of curvature for the posterior surface of the lens was larger in group 2 than that in group 1 (*P* = .002). The lens tilt was smaller in group 1 compared with group 3 (4.30 vs 4.90 degrees, *P* < .05), and the lens decentration was smaller in group 1 compared with group 2 (0.16 vs 0.21 mm, *P* < .05) and group 3 (0.16 vs 0.20 mm, *P* < .05). The distribution of sex and other preoperative biometric parameters were similar among the 3 groups (all with *P* > .05).

The median interval between PPV and cataract surgery was 7.5 months (IQR: 5.27 to 14.3 months). All eyes were implanted with a foldable IOL in the capsular bag. Specifically, 257 eyes (68.5%) were implanted with a hydrophobic IOL, and 99 eyes (26.4%) were implanted with 3-piece IOLs. At 3 months postoperatively, 134 eyes (35.7%) exhibited complete capsulorhexis–IOL overlap. The mean postoperative ACD was 4.64 ± 0.38 mm for all study participants. The median IOL tilt was 4.50 degrees (IQR: 3.40 to 6.10 degrees), and 64 eyes (17.1%) displayed clinically significant IOL tilt. The median IOL decentration was 0.22 mm (IQR: 0.12 to 0.34 mm), and 74 eyes (19.7%) exhibited clinically significant IOL decentration (Table 2). Similar to crystalline lenses, most of IOLs (70.6%) tilted toward the inferotemporal direction, and no obvious tendency was displayed in the directions of IOL decentration (Figure 1). The ACD of group 2 was larger than that of group 3 (4.73 ± 0.38 vs 4.59 ± 0.35 mm, *P* = .003). There was no statistically significant difference in IOL tilt among the 3 groups (*P* = .90), whereas a larger IOL decentration was observed in group 3 compared with group 1 (0.26 vs 0.17 mm; *P* < .001).

**Table 2.** Position of IOLs of participants

Parameter	Total	Group 1	Group 2	Group 3	*P* value
Postop ACD (mm), mean ± SD	4.64 ± 0.38	4.65 ± 0.40	4.73 ± 0.38	4.59 ± 0.35	.02*
IOL tilt (°), median (IQR)	4.50 (3.40, 6.10)	4.50 (3.40, 6.00)	4.60 (3.30, 6.40)	4.50 (3.30, 6.10)	.90
<7 (%)	311 (82.9)	107 (88.4)	64 (81.0)	140 (80.0)	.15
≥7 (%)	64 (17.1)	14 (11.6)	15 (19.0)	35 (20.0)	
IOL decentration (mm), median (IQR)	0.22 (0.12, 0.34)	0.17 (0.10, 0.27)	0.23 (0.15, 0.32)	0.26 (0.14, 0.41)	<.001*
<0.4 (%)	301 (80.3)	107 (88.4)	63 (79.7)	131 (74.9)	.02*
≥0.4 (%)	74 (19.7)	14 (11.6)	16 (20.3)	44 (25.1)	

ACD = anterior chamber depth, as measured from the corneal epithelium to IOL; IQR = interquartile range

* Statistically significant (*P* < .05)

Multiple logistic regression demonstrated that lens tilt (OR = 1.44, 95% CI, 1.21-1.71; *P* < .001), lens decentration (OR = 1.74, 95% CI, 1.32-2.28; *P* < .001), lens diameter (OR = 0.49, 95% CI, 0.28-0.85; *P* = .01), and hydrophilic IOL (OR = 2.36, 95% CI, 1.21-4.62; *P* = .01) were associated with IOL tilt over 7 degrees (Table 3). In addition, lens tilt (OR = 1.24, 95% CI, 1.07-1.44; *P* = .005), lens decentration (OR = 2.30, 95% CI, 1.76-3.00; *P* < .001), and incomplete capsulorhexis–IOL overlap (OR = 2.44, 95% CI, 1.18-5.05; *P* = .02) were significantly associated with IOL decentration over 0.4 mm.

**Table 3.** Univariate and multivariate logistic regression of clinically significant IOL tilt and decentration

Factors	IOL tilt ≥7°				IOL decentration ≥0.4 mm			
	Univariate analysis		Multivariate analysis		Univariate analysis		Multivariate analysis	
	OR (95% CI)	*P* value	OR (95% CI)	*P* value	OR (95% CI)	*P* value	OR (95% CI)	*P* value
AL (mm)	0.97 (0.86, 1.09)	.65			1.02 (0.92, 1.14)	.71		
ACD (mm)	0.43 (0.20, 0.91)	.03^a^*			0.54 (0.27, 1.08)	.08		
RAL (mm)	0.75 (0.60, 0.93)	.01*	0.81 (0.62, 1.06)	.13	0.87 (0.71, 1.06)	.18		
RPL (mm)	0.83 (0.54, 1.27)	.39			1.14 (0.79, 1.66)	.48		
Lens thickness (mm)	1.46 (0.79, 2.72)	.22			0.87 (0.48, 1.57)	.64		
Lens vault (mm)	1.48 (0.62, 3.51)	.38			0.86 (0.38, 1.93)	.71		
Lens tilt (°)	1.73 (1.49, 2.02)	<.001*	1.44 (1.21, 1.71)	<.001*	1.59 (1.38, 1.82)	<.001*	1.24 (1.07, 1.44)	.005*
Lens decentration (0.1 mm)	2.19 (1.76, 2.72)	<.001*	1.74 (1.32, 2.28)	<.001*	2.64 (2.09, 3.34)	<.001*	2.30 (1.76, 3.00)	<.001*
Lens diameter (mm)	0.47 (0.30, 0.76)	.002*	0.49 (0.28, 0.85)	.01*	0.72 (0.47,1.11)	.13		
Corneal diameter (mm)	0.61 (0.34, 1.10)	.10			0.82 (0.47, 1.42)	.48		
Diabetes mellitus	1.67 (0.90, 3.09)	.10			1.32 (0.72, 2.41)	.37		
Surgery type								
Group 1	1.00 (Ref)	-			1.00 (Ref)	-	1.00 (Ref)	-
Group 2	1.79 (0.81, 3.95)	.15			1.94 (0.89, 4.24)	.10	0.82 (0.30, 2.29)	.71
Group 3	1.91 (0.98, 3.73)	.06			2.57 (1.34, 4.93)	.005*	1.43 (0.61, 3.38)	.41
SO tamponade	1.87 (0.99, 3.54)	.054			2.36 (1.26, 4.43)	.007^a^*		
Peripheral vitreous shaving	3.53 (1.06, 11.73)	.04*	1.50 (0.42, 5.36)	.53	4.27 (1.29, 14.13)	.02*	1.46 (0.35, 6.08)	.60
Time interval between PPV and cataract surgery (y)	0.99 (0.86, 1.13)	.85			1.03 (0.91, 1.15)	.66		
Incomplete capsulorhexis–IOL overlap	1.39 (0.78, 2.49)	.27			2.57 (1.39, 4.74)	.002*	2.44 (1.18, 5.05)	.02*
IOL, hydrophilic	1.91 (0.10, 3.31)	.02*	2.36 (1.21, 4.62)	.01*	1.06 (0.61, 1.82)	.84		
IOL, 1 piece	1.90 (0.95, 3.81)	.07			0.81 (0.46, 1.42)	.47		

ACD = anterior chamber depth, as measured from the corneal epithelium to lens; AL = axial length; OR = odds ratio; RAL = radius of anterior lens surface curvature; RPL = radius of posterior lens surface curvature; SO = silicone oil

* Statistically significant (*P* < .05)

a Preoperative ACD and SO tamponade were not included in multivariate logistic regression due to the collinearity

**Figure 2.**
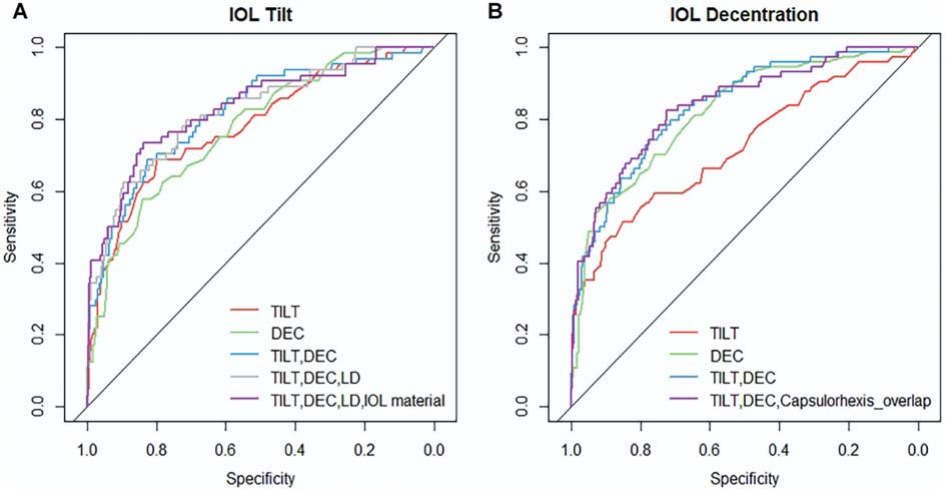
Receiver operating characteristic curves for predicting IOL tilt (A) or decentration (B) with different predictor combinations. Individual predictors are lens tilt, lens decentration, lens diameter, IOL material (hydrophobic vs hydrophilic), and capsulorhexis–IOL overlap (complete vs incomplete). DEC = lens decentration; LD = lens diameter; TILT = lens tilt

Figure 2 shows the receiver operating characteristic curve of different prediction models with different combinations of parameters based on the multiple logistic regression results. For predicting postoperative IOL tilt over 7 degrees, the model based solely on preoperative lens tilt achieved an AUC of 0.78 (95% CI, 0.71-0.85). Similarly, the model based solely on preoperative lens decentration also achieved an AUC of 0.78 (95% CI, 0.71-0.84). Prediction based on both preoperative lens tilt and lens decentration could further increase the AUC (0.82, 95% CI, 0.76-0.88; *P* < .05). Further inclusion of preoperative lens diameter (AUC: 0.82, 95% CI, 0.76-0.88) and IOL material (AUC: 0.83, 95% CI, 0.77-0.89) resulted in only slight and no statistically significant improvement (*P* > .05).

The strongest single predictor for significant IOL decentration (over 0.4 mm) was preoperative lens decentration (AUC: 0.82, 95% CI, 0.77-0.87), followed by preoperative lens tilt (AUC: 0.72, 95% CI, 0.65-0.79). Similarly, combining lens tilt and lens decentration could enhance the prediction ability (AUC: 0.84, 95% CI, 0.78-0.89), but the addition of capsulorhexis–IOL overlap made no statistically significant improvement (AUC: 0.84, 95% CI, 0.79-0.89; *P* > .05).

**Figure 3.**
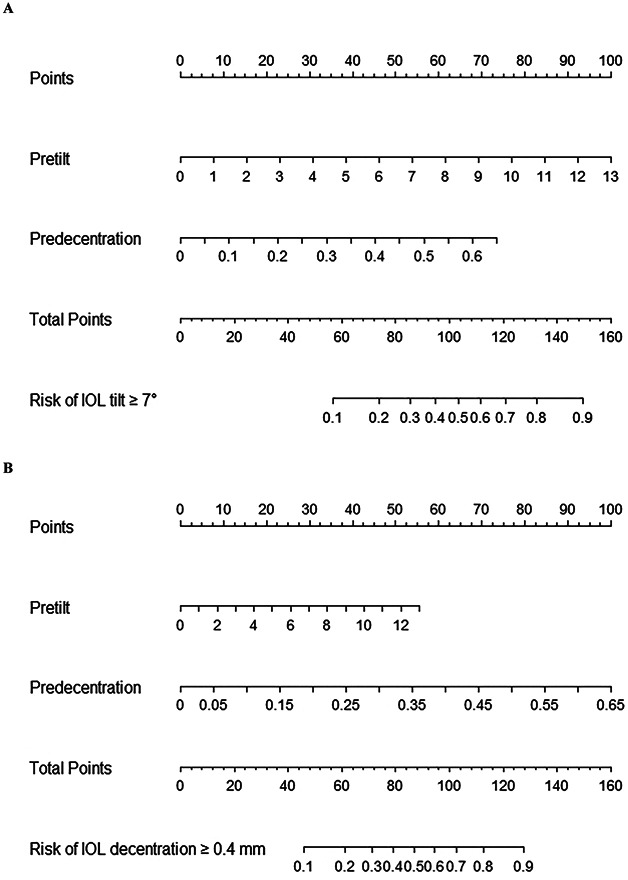
Nomogram models. *A*: IOL tilt ≥7 degrees. *B*: IOL decentration ≥0.4 mm. An example using nomogram (*A*) to estimate the probability of IOL tilt over 7 degrees for a specific patient. Draw a line vertically upward to the “Points” bar to calculate the point of preoperative crystalline lens tilt and decentration, separately. Based on the sum of the points, draw a downward vertical line from the “Total Points” line to calculate the risk.

Based on the above results, we constructed 2 nomograms that graphically depict a statistical prognostic model to generate a probability of clinically significant IOL misalignment using preoperative crystalline lens tilt and decentration (Figure 3). The magnitudes of preoperative lens tilt and decentration were listed separately, with a matching number of points allocated to each. The cumulative point score for all the factors was then matched to an outcome scale. The AUC of the nomogram model for predicting postoperative IOL tilt over 7 degrees and IOL decentration over 0.4 mm was 0.82 (95% CI, 0.76-0.88) and 0.84 (95% CI, 0.78-0.89), respectively. The calibration analysis showed a good relationship between observed and predicted events (Hosmer-Lemeshow test, *P* value was 0.46 for IOL tilt and 0.48 for IOL decentration).

## DISCUSSION

Predicting the risk of clinically significant IOL misalignment in patients with cataract with prior PPV could help surgeons make better clinical decisions for maximized surgical benefits. In this study, we found that preoperative crystalline lens tilt and decentration, hydrophilic IOL implantation, and incomplete capsulorhexis–IOL overlap were significant risk factors for clinically significant IOL tilt and decentration. Furthermore, our study provided new evidence of the strong predictive ability of preoperative crystalline lens tilt and decentration for postoperative clinically significant IOL tilt (AUC = 0.82) and decentration (AUC = 0.84) in a large sample of patients with cataract with prior PPV. We believe that these findings have important pragmatic implications for surgical considerations.

We want to call for more intentions to the high incidence of clinically significant IOL misalignment in patients with cataract with prior PPV, considering that the clinically significant IOL misalignment would greatly diminish the visual quality, especially for eyes implanted with aspheric and other optical sophisticated IOLs. For patients receiving routine cataract surgery without prior PPV, 10.72% to 12% of eyes displayed clinically significant IOL tilt, and 5% to 11.22% of eyes had clinically significant IOL decentration.^8,12^ Several studies indicated that PPV and intravitreal tamponades would increase the risk of IOL misalignment. After phacovitrectomy with air tamponade, 8% to 14.3% and 15.4% to 17% of eyes displayed clinically significant IOL tilt and decentration, respectively.^14,15^ However, there is scarce evidence regarding the IOL position for post-PPV patients who underwent phacoemulsification and IOL implantation. Our previous study showed a significantly higher risk (18.3% to 21.3%) of clinically significant IOL tilt or decentration in patients with cataract with prior PPV compared with non-PPV patients (5.77% to 6.73%).^9^ In this study, the identified proportion of clinically significant IOL misalignment (17.1% to 19.1%) was similar. This high risk of IOL misalignment may be attributable to the absence of the vitreous support, which increases the zonular instability and posterior capsule mobility, and the use of intravitreal tamponade, which pushes the lens–iris diaphragm forward and stretches the zonular fibers.^16,17^

We also assessed whether SO tamponade and the timing of SO removal (sequentially or simultaneously) would influence the IOL position. A higher proportion of clinically significant IOL misalignment was observed in group 2 (19.0% to 20.3%) and group 3 (20.0% to 25.1%), compared with group 1 (11.6%). This supports our prior finding that SO tamponade was a risk factor for greater IOL tilt and decentration in patients with prior PPV.^9^ Regarding the timing of SO removal, one previous study suggested that better refractive outcomes could be obtained when SO removal and cataract surgery were performed sequentially.^18^ We found a higher risk of clinically significant IOL decentration in group 3 than in group 2 (25.1% vs 20.3%), albeit not statistically significant. This suggests the need to reconfirm the appropriate IOL position at the end of cataract surgery and perform IOL repositioning if necessary.

The current study demonstrated that preoperative tilt and decentration of crystalline lenses had the greatest impact on the risk of postoperative IOL tilt and decentration in these patients. In addition to the preoperative position of the crystalline lens, we also identified that the risk of IOL tilt over 7 degrees increased by 1.36-fold after the implantation of a hydrophilic IOL (OR = 2.36, 95% CI, 1.21-4.62), and incomplete capsulorhexis–IOL overlap was associated with a 144% increase in the risk of IOL decentration over 0.4 mm (OR = 2.44, 95% CI, 1.18-5.05). Possible explanations could be that the hydrophilic IOL showed a later and weaker adhesion between the IOL and the capsule bag compared with hydrophobic IOLs, promoting lens epithelial cell proliferation and migration, which in turn accelerated and exacerbated anterior capsule contraction; furthermore, incomplete capsulorhexis–IOL overlap might lead to asymmetrical capsule shrinking, with subsequently larger IOL tilt and decentration.^19–21^ Effects of factors related to prior PPV, including the type of surgery and peripheral vitreous shaving, on the risk of postoperative IOL misalignment were also evaluated, which showed a nonsignificant association in the multiple regression model. Among the factors mentioned above, combining lens tilt and lens decentration could achieve a high prediction ability (AUC: 0.82 to 0.84) for clinically significantly IOL misalignment, but the addition of other factors made no statistically significant improvement.

Our findings suggest that we could predict the risk of IOL misalignment with high accuracy by simply assessing the lens tilt and decentration preoperatively using SS-ASOCT. The nomograms were developed for direct clinical reference. For high-risk patients, the following could be considered for better clinical management. First, hydrophobic IOL and complete capsulorhexis–IOL overlap are recommended. Second, caution should be taken when considering the implantation of aspheric or other optical sophisticated design, since that the optical performance would be largely compromised with IOL misalignment.^22^ Last but not the least, capsular tension ring implantation could be considered given its proven benefit on patients with cataract with prior PPV by maintaining the shape of the capsular bag, reducing anterior capsulorhexis shrinkage, and stabilizing the IOL position.^23^

The strength of this study lies in the large sample size, prospective design, and the availability of lens and IOL position assessment by SS-ASOCT. Some limitations should be noted in this study. First, the effect of gas tamponades on the IOL position was not evaluated because there has no commercial perfluoropropane in China mainland from 2016 to 2021. Second, the nomograms were constructed based on a single-center study of only Chinese patients, whether they could be applied to other regions or ethnicities needs further verification.

In summary, this study provides evidence for the significant impact of preoperative crystalline lens position on the risk of incident IOL misalignment based on SS-ASOCT imaging in patients with cataract with prior PPV. The proposed nomograms allow clinicians to assess the risk of clinically significant IOL tilt and decentration before cataract surgery for better surgical decisions.

**WHAT WAS KNOWN**

- The tilt and decentration of the IOL, especially with tilt over 7 degrees or decentration over 0.4 mm, could significantly impair visual function after cataract surgery.
- Approximately 20% of vitrectomized eyes showed clinically significant IOL misalignment.

**WHAT THIS PAPER ADDS**

- The tilt and decentration of the crystalline lens, hydrophilic IOL, and incomplete capsulorhexis–IOL overlap increased the risk of clinically significant IOL misalignment.
- Preoperative lens tilt together with lens decentration was the strongest predictor of incident clinically significant IOL misalignment.
